# Optimizing aesthetic outcomes for breast reconstruction in patients with significant macromastia or ptosis

**DOI:** 10.1016/j.jpra.2018.01.005

**Published:** 2018-02-14

**Authors:** Wojciech Dec

**Affiliations:** Department of Plastic Surgery, Lenox Hill Hospital, 100 East 77th Street, New York, NY, USA

**Keywords:** Breast reconstruction, Aesthetic breast reconstruction, Macromastia breast reconstruction, Ptosis breast reconstruction

## Abstract

**Background:**

Achieving excellent aesthetic outcomes in reconstruction of large or ptotic breasts is especially challenging. Incorporating a Wise pattern into the mastectomy design is effective in reducing the excess breast skin, however it increases the risk of mastectomy skin necrosis. The aim of this study is to describe surgical maneuvers which optimize aesthetic outcomes, anticipate flap volume requirements, and limit mastectomy skin necrosis in autologous reconstruction in patients with macromastia and grade III ptosis.

**Methods:**

This is a retrospective review of operative and clinical records of patients who underwent unilateral or bilateral breast reconstruction with autologous tissue between August 2015 and May 2017. Patients were divided into macromastia and ptosis groups. Key surgical maneuvers for safely achieving aesthetically optimal results were identified.

**Results:**

A total of 29 breasts were successfully reconstructed in 19 patients with a Wise pattern mastectomy skin reduction. Free flap weights were similar in both groups, mastectomy weights were greater in the macromastia group, p < 0.05. Complications were limited to three cases of wound breakdown and one case of mastectomy skin necrosis. Total number of revision stages was reduced in unilateral reconstructions when a contralateral breast reduction or mastopexy was performed during the first stage.

**Conclusions:**

A Wise pattern can safely and effectively be incorporated into a mastectomy incision design in patients who are not candidates for a nipple sparing mastectomy. Optimal aesthetics are achieved with similar volume flaps for both macromastia and ptosis patients. In cases of unilateral breast reconstruction a contralateral breast reduction or mastopexy should be performed at the time of the immediate breast reconstruction.

## Introduction

Breast reconstruction in the setting of significant macromastia or grade III ptosis carries an increased risk of wound healing complications, and aesthetic outcomes are often limited.^1^, ^2^, ^3^ This is especially pronounced in cases of obesity.^4^, ^5^ The surgical plan must address both replacing the breast volume as well as managing the excessive skin envelope. The application of autologous tissue flaps is well suited for this scenario.^6^, ^7^, ^8^ However, the technical difficulty of operating on an obese patient, with large volume tissue flaps, often with questionable mastectomy skin viability, and significant breast asymmetry may narrow the surgical focus to simply achieving a living flap, with aesthetic considerations relegated to a secondary goal. With careful planning and an eye for detail, excellent aesthetic breast reconstruction results are achievable for patient with macromastia or significant ptosis.^9^, ^10^, ^11^, ^12^, ^13^

This study evaluated the technique of converting a circumvertical mastectomy incision into a Wise pattern in order to reduce the risk of mastectomy skin necrosis and to achieve an optimal aesthetic appearance of the reconstructed breast in the setting of significant preoperative macromastia or ptosis. The article reviews the author's reconstructive algorithm, patient data, key technical points, and presents case studies for patients with macromastia and grade III ptosis who are not candidates for a nipple sparing mastectomy.

## Patients and methods

### Demographics

**Figure 1:**
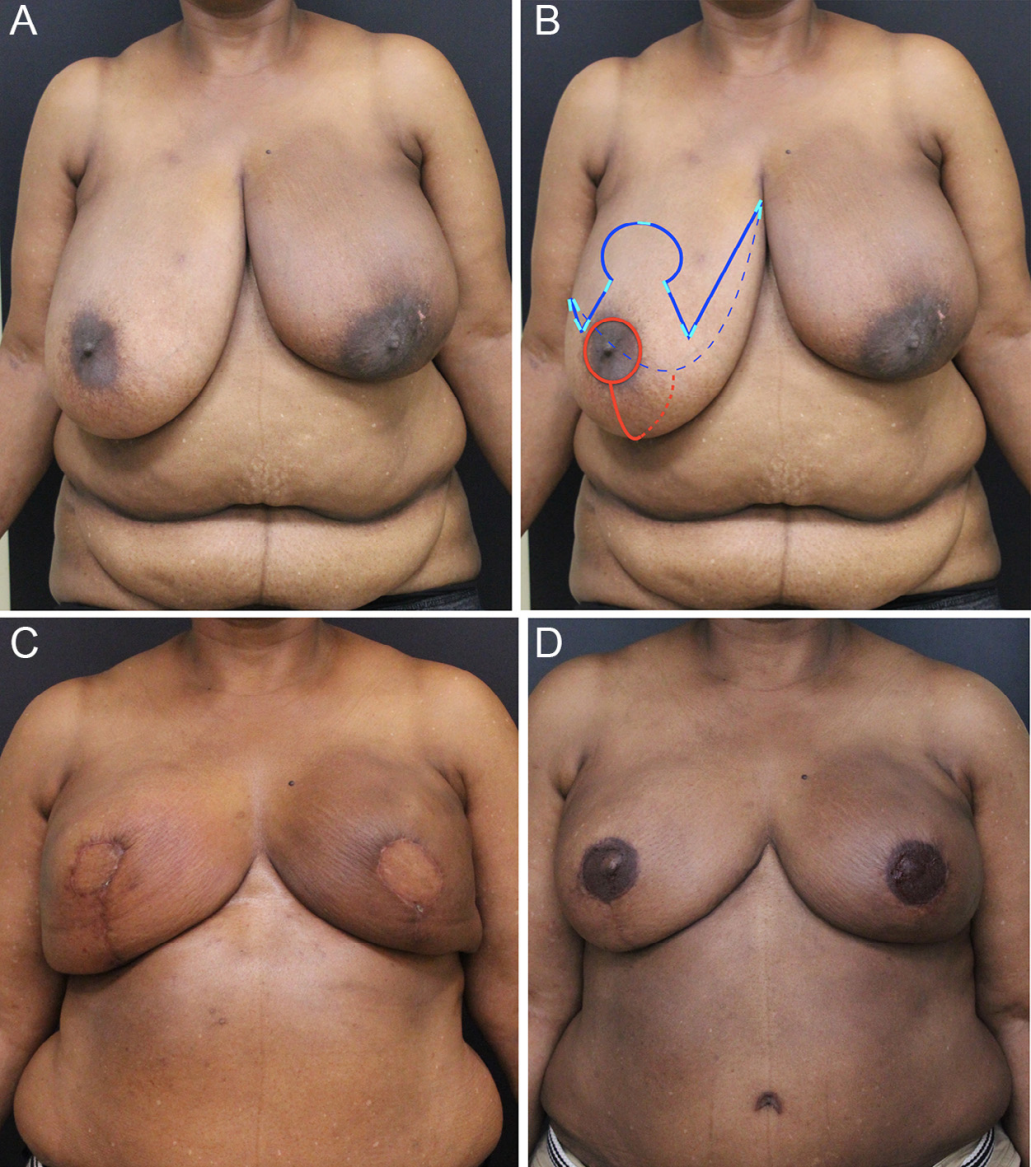
Patient with preoperative macromastia. A. Preoperative view of patient with macromastia who is indicated for a bilateral mastectomy. Left breast has previously been treated with lumpectomy and radiation therapy. B. Preoperative markings. Red markings represent circumvertical mastectomy incision. Blue markings represent Wise pattern and inframammary crease. Dotted lines represent markings hidden behind the ptotic breast. The Wise pattern is adjusted and incised after flap reperfusion and inset are complete to ensure optimal skin envelope redraping. White markings represent location of staples placed to register Wise pattern, which must be preserved through the operation. C. Following completion of the first stage of breast reconstruction with bilateral DIEP flaps, and Wise pattern skin envelope reduction. D. Following bilateral breast reconstruction revision surgery and nipple areola reconstruction and tattooing. (For interpretation of the references to color in this figure legend, the reader is referred to the web version of this article.)

**Figure 2:**
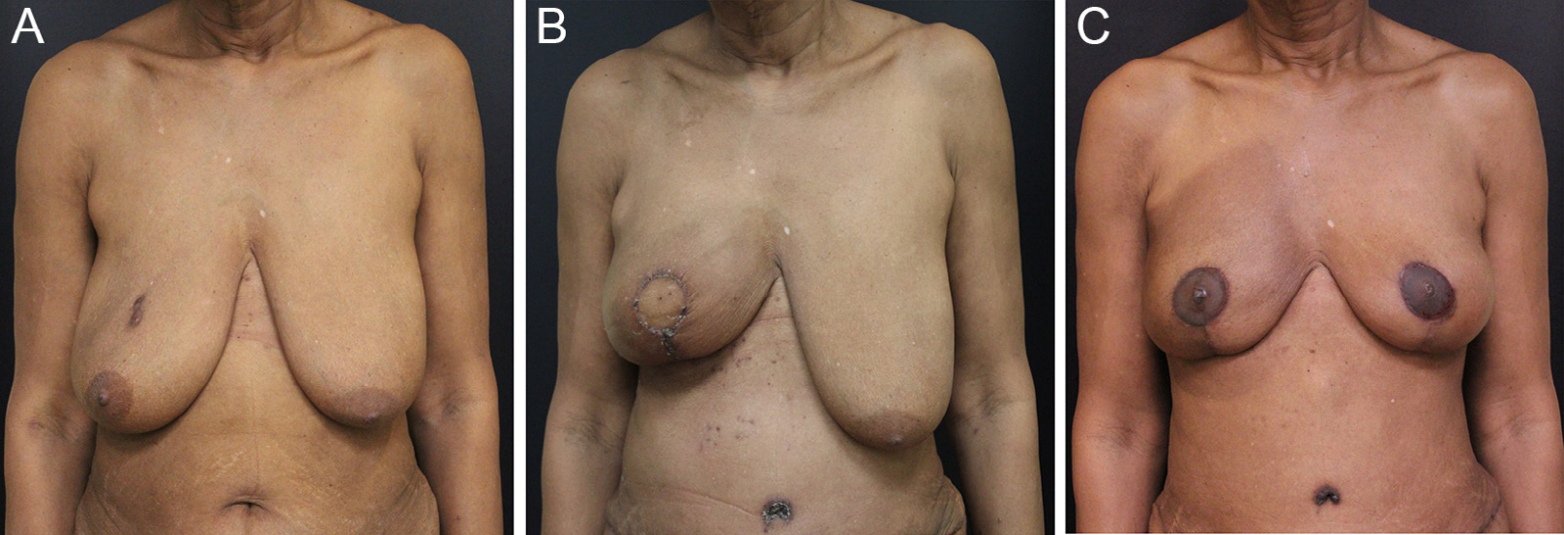
Patient with preoperative grade III breast ptosis. A. Preoperative view of patient with grade III ptosis who is indicated for a right mastectomy. B. Following completion of first stage right breast reconstruction with stacked DIEP flaps, and Wise pattern skin envelope reduction. The left breast remains untreated. C. Following right breast reconstruction revision surgery and nipple areola reconstruction and tattooing, right breast radiation therapy, and left breast mastopexy for symmetry. The natural left nipple areola has also been tattooed to achieve exact color match between breasts.

A retrospective review was conducted in 19 consecutively presenting patients with macromastia or grade III ptosis who underwent 29 autologous unilateral or bilateral breast reconstructions performed by W.D. from August 2015 to May 2017 (Table 1). Ten patients who underwent sixteen free flap breast reconstructions were classified as having macromastia based on the Schnur sliding scale and the difference between the mastectomy and flap weights (Figure 1). In this group four patients underwent unilateral reconstruction and six patients underwent bilateral reconstruction. The remaining nine patients who underwent thirteen free flap breast reconstructions were classified as having grade III breast ptosis (Figure 2). Five of these patients underwent unilateral reconstruction and four patients underwent bilateral reconstruction.

**Table 1:** Breast reconstruction patient data.

	Macromastia	Ptosis
Number of patients (19)	10	9
Mean patient age in years (50.5)	47.6	53.7
Mean patient BMI (29.3)	30.4	28.1
Number of flaps (29)	16	13
Unilateral reconstructions (9)	4	5
Bilateral reconstructions (10)	6	4
Mean mastectomy weight in grams (938)	1183*	636*
Mean flap weight in grams (598)	589	609

* Denotes a statistically significant difference between values, p < 0.05.

Data regarding patient characteristics, treatment history, details of surgical interventions, and photographs were collected.

### Breast reconstruction technique

All patients reviewed in the study underwent autologous breast reconstruction with DIEP (26) or PAP (3) flaps. PAP flaps were performed in two patients who had previously undergone an abdominoplasty. Fourteen patients underwent second stage revision surgery, which involved fat grafting, adjustment of the skin envelope, nipple reconstruction, and donor site revisions. Nipple reconstruction was performed using the CV flap technique. Fat harvest was performed using tumescent technique with a 4 mm basket cannula without power assistance. Fat was processed with the aid of the Revolve system and aliquoted into 10 cc syringes. Adherent scars, such as those which frequently occur in the area of the axilla were released by subcision using an 18 gauge hypodermic needle. Fat injection was performed with a high number of cannula passes to limit fat clumping using 18 gauge single port microcannulas.

### Statistical analysis

A two tail Student t-test was used to compare mastectomy and flap weights between patients. A value of p < 0.05 was used to determine statistical significance.

## Results

Nineteen consecutively presenting patients with preoperative macromastia or grade III ptosis who underwent unilateral or bilateral autologous breast reconstruction were included in the study. The average patient age was 50.5 years old (range 25–68). The mean mastectomy weight was 1183 g (range 885 g–1855 g) in the macromastia group, and 636 g (range 338 g–1062 g) in the ptosis group; this difference was statistically significant. The mean free flap weight was 589 g (range 380 g–760 g) in the macromastia group and 609 g (range 431 g–830 g) in the ptosis group.

Nine patients in the study underwent unilateral breast reconstruction and contralateral breast reduction or mastopexy. The mean breast reduction and skin excision weight was 488 g in the macromastia group, and 114 g in the ptosis group (p < 0.05).

No flaps were lost; complications were limited to wound healing complications secondary to incision breakdown around the “T” point or vertical limb, which occurred in three patients (2 macromastia, 1 ptosis) and did not require return to the operating room. One patient (macromastia) experienced mastectomy skin necrosis. The area of mastectomy skin necrosis measured 4 × 4 cm and occurred along the vertical limb of the Wise pattern. The wound was debrided under local anesthesia and allowed to heal secondarily. Two of the patients with a wound healing problem suffered from obesity and diabetes, one of whom was a former smoker. The third patient with a wound healing complication had macromastia without obesity and no other predisposing factors. The one patient who experienced mastectomy skin necrosis suffered from macromastia and obesity.

Fourteen patients underwent a second stage breast reconstruction revision which included nipple reconstruction, fat grafting, and modifications of the skin envelope, as well as modification of the donor sites. Two patients in the group underwent a second revision (third operation) to optimize breast symmetry. Both were unilateral breast reconstruction cases where no symmetry procedure was performed at the time of the mastectomy. One of these patients was in the macromastia group and one was in the ptosis group. The remainder of the unilateral breast reconstruction patients underwent a symmetry procedure at the time of their mastectomy, and only required a single fine adjustments revision for achieving breast symmetry, thus limiting the reconstruction to a total of two operations.

## Discussion

The surgical approach to patients with macromastia and grade III ptosis is similar. Although the mastectomy weights differ considerably between the two groups (1183 g vs. 636 g, p < 0.05) flap weights are similar (589 g vs. 609 g). Likewise no statistical difference was noted in adverse events between the two groups. The mastectomy skin flaps are protected by performing the mastectomy through a circumvertical incision (Figure 1B, red markings). The Wise pattern is incised only when the free flap is reperfused and inset. This strategy protects the mastectomy flap skin edges from traction or crush injury. Additionally the mastectomy skin envelope can be precisely adjusted to redrape over the free flap. The preoperative Wise pattern markings merely act as a reference frame and may be adjusted based on tissue requirements. In all cases the freshly cut skin edges were observed to have punctate bleeding, and no additional diagnostic intervention, such as SPY, was required. The practice of utilizing optimally tailored mastectomy skin to achieve a tension free closure has resulted in only one case of mastectomy skin necrosis.

### Managing the contralateral breast in unilateral reconstruction

Unlike bilateral breast reconstruction, achieving breast symmetry in unilateral breast reconstruction cases requires additional consideration. A contralateral breast reduction or mastopexy procedure is always required in this setting. In all cases two operations, a coarse adjustment and a fine adjustment, were required to symmetrize the healthy breast to the reconstructed breast. Factors related to breast volume, nipple position, intraoperative edema, postoperative tissue drop create a complex scenario of multiple moving pieces that makes accurate predictions about final symmetry very difficult. For this reason a symmetry procedure should be initiated at the time of the mastectomy to minimize the total number of stages to complete the reconstruction. Patients for whom the contralateral breast was not addressed at the time of the mastectomy required a total of three operations (**1**. unilateral breast reconstruction; **2**. breast reconstruction revision, nipple reconstruction, and contralateral course adjustment symmetry procedure; and **3**. fine adjustment symmetry procedure) compared to two operations in patients who underwent a symmetry procedure at the time of their mastectomy (**1**. unilateral breast reconstruction and contralateral course adjustment symmetry procedure; and **2**. breast reconstruction revision, nipple reconstruction, and fine adjustment symmetry procedure).

In macromastia patients at the time of the coarse adjustment symmetry procedure the difference between the mastectomy weight and the flap weight can guide the extent of the contralateral reduction.

### Flap monitoring

**Figure 3:**
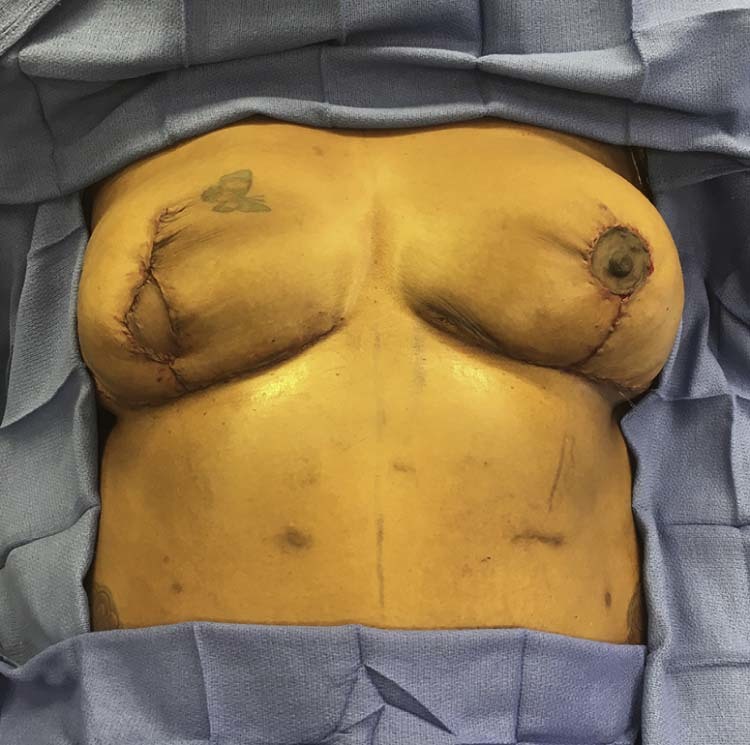
Intraoperative view of patient following right mastectomy and breast reconstruction with a PAP flap, Wise pattern breast skin reduction, and left breast reduction for symmetry. The PAP flap skin paddle is externalized along the vertical limb of the Wise pattern with a plan for excision at the second stage operation.

It is the author's preference to monitor the flap with a skin paddle. In all patients who underwent DIEP flap reconstructions in this study a small elliptical skin paddle was externalized to be later incorporated into a nipple areola reconstruction. For PAP flap cases a vertically oriented skin paddle was externalized within the vertical limb of the Wise pattern (Figure 3). In cases where no Doppler signal is captured over the externalized portion of the flap, an implantable Doppler is used to monitor the arterial portion of the flap, and the flap skin paddle color is monitored to assess the venous outflow. This is preferred to leaving an excessively large or poorly positioned skin paddle in order to capture a skin perforator signal. Following completion of nipple areola reconstruction in DIEP flap patients the skin paddle should fall entirely within the area of the tattooed areola (Figure 1, Figure 2). For PAP flap patients the skin paddle is simply excised.

### Nipple reconstruction and additional procedures

A second stage breast reconstruction revision operation is recommended to all patients to be performed at the time of the nipple reconstruction. During this stage the focus is on optimizing aesthetics and symmetry, by performing nipple reconstruction, fat grafting, and adjusting the skin envelope. Although nipple sparing mastectomy is technically feasible in patients with macromastia or grade III ptosis, there is little aesthetic advantage in performing this procedure for this group of patients, especially when weighed against the increased risk of wound healing complications.^14^, ^15^ For this reason it is the author's preference to perform a formal nipple areola reconstruction in patients with macromastia or grade III ptosis. Nipple reconstruction is performed by utilizing CV flaps within the DIEP flap skin paddle. The eventual nipple areola tattoo overlaps the scars from the nipple reconstruction resulting in a scarless appearance of the reconstructed nipple areola (Figure 1, Figure 2). For PAP flap patients the skin paddle is only used for monitoring and is excised; the nipple reconstruction is performed with mastectomy skin.

Fat grafting is commonly utilized at the time of the secondary procedure. Fat graft is used to disguise surface contour irregularities, which commonly occur along the perimeter of the flap. In the case of a significant breast asymmetry fat graft is placed diffusely into the breast flap to increase its volume. The abdominal donor site frequently benefits by obtaining the fat graft from areas of excessive fullness.

Additional modifications of the breast skin envelope are frequently required. This is consistently true in the inferolateral aspect of the breast where an unsightly skin excess exists along the transition from the inframammary fold to the lateral chest wall (compare Figure 1C and 1D). This is easily corrected with a spindle shaped skin excision.

## Conclusions

Patients with macromastia or grade III ptosis without macromastia can be treated with the same surgical approach. Despite different mastectomy weights, flap weights required to complete the reconstruction are similar. A Wise pattern can safely and effectively be incorporated into a mastectomy incision design. A limited circumvertical incision is initially marked for the mastectomy, and the final Wise pattern is incised only after completion of the reconstruction. This has the advantage of tailoring the breast skin surface area to optimally match the flap volume, as well as protect the mastectomy flap skin edges from inadvertent tissue injury from retraction. In cases of unilateral breast reconstruction a contralateral breast reduction or mastopexy should be performed in the immediate setting; this practice affords an opportunity to further fine tune breast symmetry discrepancies in a single revision procedure.

## Conflict of interest

No financial support or benefits have been received by the author from any commercial source which is related directly or indirectly to the scientific work reported in the article.
